# National Pharmacare in Canada: Equality or Equity, Accessibility or Affordability Comment on "Universal Pharmacare in Canada: A Prescription for Equity in Healthcare"

**DOI:** 10.15171/ijhpm.2019.146

**Published:** 2020-01-01

**Authors:** Nigel S. B. Rawson

**Affiliations:** ^1^Eastlake Research Group, Oakville, ON, Canada.; ^2^Canadian Health Policy Institute, Toronto, ON, Canada.; ^3^Fraser Institute, Vancouver, BC, Canada.

**Keywords:** Universal Pharmacare, Health Policy, Specialized Care Drugs, Accessibility, Equity

## Abstract

Canada’s federal government intends to take steps to implement national pharmacare so that all Canadians have prescription drug coverage they need at an affordable price. Relatively limited funds have so far been pledged to support national pharmacare, which raises the question: what kind of program is envisioned? Since the government has already introduced regulations intended to reduce new drug prices drastically, national pharmacare seems likely to be a basic system designed to assist low-income Canadians with accessing primary care medicines. What Canadians actually need is a system that provides access to the medicine considered appropriate by the patient and their healthcare provider for the patient’s specific condition. Equitable national pharmacare will not be achieved if patients are denied access to new high-cost specialized medicines that can improve or extend their lives, any more than if patients who cannot afford basic drugs are not helped.

Two recent *IJHPM* articles focused on issues around the creation of national pharmacare in Canada. Lewis^1^ discussed issues in establishing a single-payer government drug insurance program, while Hajizadeh and Edmonds^2^ examined existing differences in out-of-pocket expenditures on pharmaceuticals. Both articles raise important concerns.

## Government Insurance for Prescription Medicines

 Canada has a universal government healthcare insurance scheme that covers physician, hospital (including medicines administered there) and laboratory services but not medicines dispensed in the community. Federal, provincial and territorial (FPT) governments have prescription drug plans that offer a degree of coverage to about a quarter of the population comprising seniors, social assistance recipients and some special groups such as cancer patients or when costs are deemed catastrophic.

Several barriers must be overcome by pharmaceutical manufacturers to have a drug listed on government formularies, the first being health technology assessment (HTA). To be considered for coverage in all government plans, except those in Quebec, manufacturers submit an HTA application to the Canadian Agency for Drugs and Technologies in Health (CADTH) to demonstrate the medicine’s value based on its clinical benefit relative to its cost (Quebec has its own HTA agency). Lewis praised CADTH as having “excellent drug assessment expertise,”^1^ but ignores the fact that CADTH is owned and funded by FPT health ministries and, consequently, does not operate at arms-length from them. This results in its processes failing to adhere to good governance principles, particularly accountability, transparency, and stakeholder participation,^3^ which has led to criticism from patients and others, especially regarding how CADTH assesses high-cost rare disorder drugs.^4,5^

Following HTA, manufacturers usually seek admission to the FPT governments’ collective price negotiating process, known as the pan-Canadian Pharmaceutical Alliance (pCPA), whose main objective is to capitalize on the combined governments’ buying power. Each provincial drug plan decides at the beginning of a negotiation whether to be included. If a negotiation is successfully completed, the cost and criteria under which FPT governments will pay for the drug are agreed. CADTH and the pCPA are closely connected so that, in general, a negative HTA recommendation results in no negotiation and a positive one sets up negotiating factors, usually the need for a substantial price reduction.^6^ A successful pCPA negotiation does not guarantee that all FPT plans will cover the medicine because an individual agreement must be reached with each plan because drug coverage is a provincial decision. Since the process is confidential, it is not known whether a drug is not listed by a drug plan because the province opted out of the negotiation, or whether it opted in but failed to complete an agreement with the manufacturer.

Moreover, coverage does not mean that patients necessarily have easy and inexpensive access. FPT government plans have complex systems of deductibles, copayments and premiums and, for many medications, restricted access criteria that result in variation in patient eligibility, out-of-pocket expenses and coverage, which has led to inequalities in what drugs are covered, who gets access, and out-of-pocket costs between plans.^7^ Hajizadeh and Edmonds^2^ see national pharmacare as a way to reduce variation in out-of-pocket expenditures.

## Private Insurance for Prescription Medicines

Coverage of medicines is also available through private insurance paid for by individuals themselves or cost-shared with their employers, unions or associations. Over two-thirds of Canadians have access to private insurance through a range of plans from those that offer relatively limited drug coverage to those that cover virtually all medicines with regulatory approval, the extent of each plan’s coverage being determined by how much clients are willing to pay in premiums. Some private plans have lifetime caps of a set amount, meaning that the company will only cover a high-cost drug for a few years after which the patient is faced with paying or not having the medicine.^8^ Insurance companies take note of HTA recommendations but do not necessarily base coverage on them.

Private insurers are thought to pay higher prices than government plans, which is one reason why Lewis believes private insurance plans are inefficient and should be replaced by a national government plan.^1^ Another reason is that only a small proportion of all new drugs offer substantial therapeutic benefits over existing medicines, although innovative drugs for rare diseases for which there are no existing therapies are likely to provide much greater benefit. Nevertheless, many Canadians rely on private insurance to access medicines they would otherwise be unable to due to cost or delays in government systems. For example, in an open letter to the Prime Minister, a cystic fibrosis sufferer, wrote: “I had a health crisis and was on the verge of expiring and requiring a lung transplant in 2012. I received access to the drug Kalydeco and my lung function bumped from 30% to 50% and has stayed at 50% since 2012. If I did not have an employer benefit plan and was forced to wait until Kalydeco was approved in 2014, I would have likely died well before the drug was approved for public reimbursement.”^9^

 A federal government seeking to replace private insurance with single-payer government-funded national pharmacare providing significantly less comprehensive coverage would be extremely unpopular.

## Equality or Equity?

In the belief that national pharmacare would reduce inequalities in existing government programs, Lewis^1^ and Hajizadeh and Edmonds,^2^ like many other Canadian academics, politicians, government officials and patients, want pharmacare incorporated within the existing healthcare system, although Lewis recognizes that stakeholder interests in keeping the status quo will present challenging difficulties. Equality means treating everyone exactly the same way. The risk of a system focused solely on equality is that it could just as well equally deny access to medicines to all patients as equally provide access. Significant improvements in equality could be realized within the present government drug plans without the need to replace them with an expensive new initiative. For instance, governments could eliminate deductibles and copayments for low-income Canadians or make clinical criteria required to obtain coverage for many drugs consistent in all plans.

National pharmacare should be focused on equity, ie, it should be a fair and just system delivering medicines for everyone to benefit based on their need.^10^ Although it may be a difficult and complex task to achieve, the objective should be equity-based national pharmacare that would provide the medicine deemed appropriate by the patient and their healthcare provider for the patient’s specific condition. This type of program would not only provide coverage to patients who presently have none but would also extend appropriate coverage to patients whose insurance, whether government or private, denies access due to a drug’s high cost or limits access by requiring the patient’s circumstances to match clinical criteria that are overly restrictive or that make little clinical sense. This type of program would satisfy present unmet needs and be more likely to lead to appropriate prescribing and improved health outcomes, which should be a primary goal of all governments. As Lewis noted, Canada “can afford a generous program,”^1^ but will the federal government opt for a generous one or miserly one?

## Affordability or Accessibility?

Canada’s federal government promotes “affordability, accessibility and appropriate use of prescription drugs,”^11^ but affordability seems to be the main objective since drug costs in Canada are second only to the United States. The government sees the road to achieving accessibility as one in which affordability is attained by introducing new far-reaching powers for the tribunal that sets price controls on new medicines. It intends to reduce the prices of new high priority medicines by “40% on average”^12^ which, in practice, means medicine developers may be required to drop prices by up to 70%, perhaps more.^13^ A reasonable reduction should be manageable, but 40%-70% is an unsustainable business model that will lead to manufacturers long delaying the launch of innovative medicines in Canada or not bringing them at all. Appropriate drug use is unattainable if Canadians cannot access new medicines because manufacturers avoid Canada due to punitive price controls.

 Many politicians, government officials and academics in Canada believe that, because pharmaceutical manufacturers seek regulatory approval in Europe before Canada and launch their products at lower prices than in Canada, introducing dramatically reduced drug prices in Canada will have little impact on how companies view the attractiveness of Canada as a market for their medicines. They ignore the facts that many western European countries present larger markets than Canada and have either pharmaceutical company headquarters or major research and manufacturing facilities within their borders. All brand name companies in Canada are affiliates of manufacturers whose headquarters and main facilities are based in other countries.

Canada is not in a strong position to demand major reductions in drug prices. A large survey of multi-national pharmaceutical executives in 31 markets performed in 2017 drew attention to the fact that stiff price cuts levied against innovative drugs hamper a country’s ability to secure and sustain investment, and in particular noted that this should be a “red flag” to economies considering a similar approach “such as Canada in its proposed amendments” to the pricing tribunal.^14^ More recently, a preliminary report of a survey of global and Canadian pharmaceutical executives performed for Life Sciences Ontario about the new pricing regulations reported that 91% of the respondents foresee “no launch” decisions being made for Canada and 96% foresee delayed launches in Canada.^15^

The 2017 survey of multi-national pharmaceutical executives reported that the pricing and reimbursement environment in New Zealand is highly damaging to innovators and the central factor undermining investment, with the use of direct price cuts and a narrow understanding of cost and savings particularly dissuading investment.^14^ Despite Canada having a larger population than New Zealand, enforcing drastically reduced drug prices in Canada will move the attractiveness of the country as a pharmaceutical market closer to the situation in New Zealand, a country whose price controls Lewis admires.^1^ New Zealand’s insurance program covers only a few drugs in each class and provides little access to new high-cost innovative medicines, including anti-cancer medications and drugs for rare disorders. When new drugs are listed, there is frequently a long delay.^16^ Pharmaceutical manufacturers seek regulatory approval for new drugs later in New Zealand than in Canada and, if they are not approved for insurance coverage, which many are not, they make them unavailable, thus reducing New Zealanders’ accessibility.^17^ While some indicators of public health are similar between Canada and New Zealand, New Zealanders with cardiovascular disorders, cancer and several other disease conditions have worse health outcomes in terms of disease survival than Canadians,^18,19^ although it is difficult to assess with confidence that reduced drug access plays a role in these differences.

In their recent election platform, the Liberals, who now form Canada’s federal government, promised a down payment of $6 billion to be shared among national pharmacare and other public health initiatives. Although $6 billion is a lot of money, it is just a drop in the bucket when compared with the $40 billion cost for pharmacare estimated by the government’s own Advisory Council on the Implementation of National Pharmacare (although some savings were anticipated),^20^ or the $48 to $52 billion projected by a tax consulting company for the Canadian Taxpayers Federation.^21^

 Despite mentioning high-cost specialized care drugs, the Advisory Council recommended pharmacare be launched by covering a short list of priority essential medicines and cited two potential starting points for creating the list: the World Health Organization’s Model List of Essential Medicines, which includes about 450 drugs intended as a formulary for developing countries, and a list of 125 primary care drugs developed by a group of Toronto clinicians. Neither list includes high-cost specialized care drugs.

Medicines for common illnesses are essential and low-income Canadians should not have to choose between paying for them or other life necessities. Nevertheless, innovative drugs for formerly untreatable disorders that save lives or significantly improve life quality are also vital. These new drugs can cost many thousands of dollars per year and frequently require life-long use, but without private or government insurance, they are unaffordable irrespective of whether a Canadian’s income is low, medium or high. It is these drugs with which provincial government premiers, who are unenthusiastic about a national program, want help from the federal government.^22^

## Conclusion

 The primary aim of national pharmacare must not be confined to cost-containment but should be to ensure the best medicines are made accessible without excessively restrictive or nonsensical clinical criteria so that patients receive the most appropriate medicine depending on their individual situation. Equity-based national pharmacare will not be achieved if patients are denied access to new high-cost specialized medicines that can improve or extend their lives, any more than if patients who cannot afford basic primary care medicines are not helped.

## Ethical issues

 Not applicable.

## Competing interests

 In the past three years, the author has received consultant fees from Advocacy Solutions, Cohn & Wolfe, and Janssen Canada Inc., research and publication fees from Bayer Inc., the Canadian Health Policy Institute, the Fraser Institute, Medicines New Zealand, Merck Sharp & Dohme (New Zealand) Ltd, RAREi (a collaboration of innovative pharmaceutical companies focused on the development of medicines for rare disorders), and Ward Health, publication processing expenses from BIOTECanada, Canadian PKU and Allied Disorders Inc., and Shire Pharma Canada ULC, and honorarium and compensation for travel from La Fondation DEVENIR. No funding was received for this work.

## Author’s contribution

 NSBR is the single author of the paper.
